# Dimension Reduction Aided Hyperspectral Image Classification with a Small-sized Training Dataset: Experimental Comparisons

**DOI:** 10.3390/s17122726

**Published:** 2017-11-25

**Authors:** Jinya Su, Dewei Yi, Cunjia Liu, Lei Guo, Wen-Hua Chen

**Affiliations:** 1 Department of Aeronautical and Automotive Engineering, Loughborough University, Loughborough LE11 3TU, UK; D.Yi@lboro.ac.uk (D.Y.); C.Liu5@lboro.ac.uk (C.L.); W.Chen@lboro.ac.uk (W.-H.C.); 2 School of Electrical Engineering and Automation, Beihang University, Beijing 100191, China; lguo@buaa.edu.cn

**Keywords:** feature extraction/selection, image classification, Hyperspectral image, PCA, SVM

## Abstract

Hyperspectral images (HSI) provide rich information which may not be captured by other sensing technologies and therefore gradually find a wide range of applications. However, they also generate a large amount of irrelevant or redundant data for a specific task. This causes a number of issues including significantly increased computation time, complexity and scale of prediction models mapping the data to semantics (e.g., classification), and the need of a large amount of labelled data for training. Particularly, it is generally difficult and expensive for experts to acquire sufficient training samples in many applications. This paper addresses these issues by exploring a number of classical dimension reduction algorithms in machine learning communities for HSI classification. To reduce the size of training dataset, feature selection (e.g., mutual information, minimal redundancy maximal relevance) and feature extraction (e.g., Principal Component Analysis (PCA), Kernel PCA) are adopted to augment a baseline classification method, Support Vector Machine (SVM). The proposed algorithms are evaluated using a real HSI dataset. It is shown that PCA yields the most promising performance in reducing the number of features or spectral bands. It is observed that while significantly reducing the computational complexity, the proposed method can achieve better classification results over the classic SVM on a small training dataset, which makes it suitable for real-time applications or when only limited training data are available. Furthermore, it can also achieve performances similar to the classic SVM on large datasets but with much less computing time.

## 1. Introduction

Recent advances in remote sensing technology make the simultaneous acquisition of hundreds of spectral bands for each image pixel a reality. The augmented image is termed Hyperspectral image (HSI), which in comparison with the conventional Red-Green-Blue (RGB) image and Multispectral image (MSI), can provide much higher spectral resolutions. This can be attributed to the increased number of bands and the decreased bandwidth of each spectral band. Consequently, a better discriminating ability is enabled in HSI, particularly for objects with similar spectral signatures in conventional images. As a result, they are attracting increasing attention in various thematic applications including ecological science (e.g., biomass estimation, land cover change detection) [1] and precision agriculture [2,3,4] (e.g., crop parameter estimation such as Leaf Area Index (LAI) and biomass, and crop health evaluation including drought, disease, grass and nutrition mapping).

In aforementioned applications, an image classification process is usually involved to convert raw image data into higher-level meaningful information. For example, in land cover classification, image pixels should be classified into different classes such as river, road, grass [1,5]; while in precision agriculture, the field should be spatially divided into classes such as normal crop and abnormal crop under various stress levels caused by drought, disease or pest. In HSI, an individual pixel is usually represented as a vector, where each entry is a measurement corresponding to a specific band. Hence, the size of pixel vector equals to the number of spectral bands. The pattern/feature vector is usually extracted from the HSI pixel vector and further treated as an instance of data sample for training and classification in pixel-wise remote sensing image classification [6].

HSI classification is never an easy task. In comparison with conventional images (e.g., RGB or MSI), the main challenges in HSI classification are caused by the high spectral dimensionality. Instead of having a few spectral bands as in RGB and MSI, there may have several hundreds of spectral bands of the same scene in HSI [7]. The increased spectral dimension makes classification model parameter estimation very difficult. First the number of parameters to be estimated increases with the increased band dimension. Second the rate of convergence of statistical estimation algorithms decreases [8]. Besides, extracting the right features in HSI classification is crucial because non-representative features can be harmful to the learning process as they require much more data to train [9].

It is well acknowledged in machine learning that in achieving a successful classification, the following factors play the dominant rules:

(a) Well defined training samples: the training data should well represent the data to be classified;
(b) Feature determination: features should maximally reflect the data information while with an appropriate dimension;
(c) Appropriate classification algorithms: the adopted algorithms should accommodate the volume of training data and the dimension of pattern vector.

Quite often, only a limited size of training dataset is available in HSI classification. This is because getting the ground truth data is labour intensive, expensive or time-consuming [10]. Usually field work for collecting data with instrumentation or even expensive laboratory tests are required with careful experiment setting or necessary environment control (e.g., moisture of soil or vegetation) [7]. Therefore reducing the reliance on large training dataset would significantly reduce the cost and time, and so increase the applicability and availability of HSI based remote sensing for a wide range of applications. In this paper, we assume a limited number of training data are given for HSI classification.

The Support Vector Machines (SVM) is chosen as the baseline algorithm for HSI classification. Its effectiveness has been validated in the area of remote sensing (see [7,11] for a comparison against other algorithms such as neural network, multinomial logistic regression, random forest and deep learning). This is mainly because in comparison to other classifiers (e.g., parametric classifiers or deep learning), SVM generalizes well even with small training samples. Consequently, this paper is mainly focussed on how to appropriately define the feature vector for pixel-wise HSI classification.

**Figure 1:**
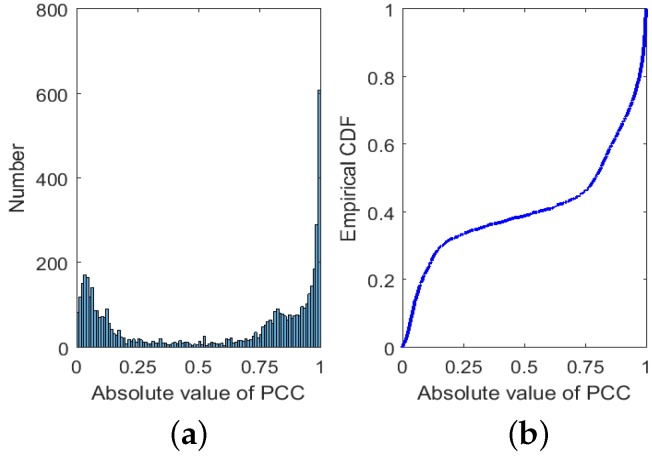
Histogram (**a**) and empirical CDF (**b**) of the absolute value of PCCs between different bands.

Engineering a good feature space is a prerequisite to achieve a high performance in many machine learning tasks. In pixel-wise HSI classification, it is a common approach to treat all bands as features and leave the problem of identifying the useful feature sets to the learning model. This simplistic approach does not always work well, particularly with a large number of bands and a comparatively small training dataset [12]. In classification tasks with high dimensional data, it is acknowledged that feature extraction and selection is of vital importance since in such applications many irrelevant (e.g., features with low correlation and noises) and redundant variables exist and comparably few training examples are available [13]. The rationales for conducting feature selection and extraction for HSI classification are summarized as follows:

(1) There are generally hundreds of bands in HSI (although not all of them are useful) while only a limited number of training samples are available;
(2) There may be a high correlation between adjacent bands [14], resulting in redundant features (see, Figure 1);
(3) Certain bands are dominated by sensor noises or may be not relevant to specific features so may contain little useful information for a task of concern.

Feature selection and extraction is a process to identify those features (feature selection) or their combinations (feature extraction) in dataset that are most useful/relevant for the predictive model construction. By doing so, the unneeded, irrelevant and redundant variables can be removed and thus providing cost-efficient (due to reduced dimensionality) predictors with a good or even better predictive accuracy while requiring less training data [15]. Besides, in certain applications such as agricultural crop monitoring, the most relevant bands can also be pinpointed using feature selection algorithms [16], providing better understanding for crop growing status. As a result, feature selection and feature extraction are drawing increasing research interest in various applications [15,17,18] including HSI analysis [13,19,20]. It was shown in [21] that the accuracy of SVM classifier could be further increased by using data dimensionality. This idea was recently validated in HSI classification where different feature selection algorithms are compared [22]. In addition, two types of feature extraction approaches were adopted in [23] for agricultural land use classification using airborne HSI. However, there is still a lack of research comparing different feature selection and feature extraction algorithms using a limited number of training samples in the context of HSI classification. Moreover, little research has been done to quantitatively investigate the computation advantages of dimension reduction aided classifiers.

The objective of the paper is to investigate the advantages of incorporating dimension reduction techniques into classifiers (e.g., SVM) for HSI classification with a limited number of training samples. To this end, several feature selection techniques including Mutual Information (MI) and Minimal-Redundancy-Maximal-Relevance (MRMR) and feature extraction techniques including Principal Component Analysis (PCA) and Kernel PCA are elaborated and compared using real HSI datasets. To be more exact, the contributions of the paper are summarized as follows:

(1) Different dimension reduction techniques including feature selection and feature extraction algorithms are compared for HSI classification, where the most suitable one is identified, namely SVM with PCA.
(2) Comparatively experimental results on a real HSI dataset demonstrate that by augmenting SVM with dimension reduction techniques (i.e., PCA), good or even better classification performance can be achieved, particularly when the size of training data is small.
(3) More importantly, it is discovered that reducing feature dimension can substantially simplify the SVM models and so reduce classification time while preserving performance, which is vital for real-time remote sensing applications.

## 2. Problem Formulation and Motivations

This part focuses on the problem formulation and research motivations. Specifically, the dataset is first introduced in Section 2.1. Then the problem of HSI image classification is formulated in Section 2.2, where the baseline classification tool, SVM, is also briefly introduced. Finally, band analysis is performed in Section 2.3, on which basis, motivations of the paper are highlighted.

### 2.1. HSI Dataset

**Figure 2:**
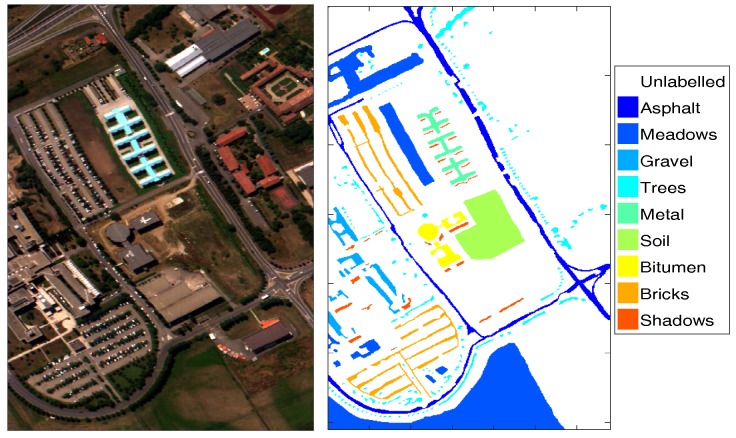
Three-band colour red (band No. 52), green (band No. 30), blue (band No. 10) composite of HSI for the University of Pavia (**left**); ground truth map (**right**): white area is not labelled.

The algorithms in this paper are validated based on one of the well known hyperspectral datasets, i.e., ROSIS urban data over Pavia, Italy. The subset capturing an urban area surrounding the University of Pavia is adopted (available http://www.ehu.eus/ccwintco/index.php?title=Hyperspectral_Remote_Sensing_Scenes), which has been applied in many previous research works [7,24,25]. The data were recorded by the ROSIS03 sensor in 2003. There are total 103 bands in this image of size 610 × 340 = 207,400 with a spatial resolution of 1.3 m per pixel and a spectral coverage range from 430 nm to 860 nm in the subset. There are nine different classes of interest (see, Figure 2); the three-band colour composite of the image and corresponding ground truth (totally 42,776 samples) map with different classes are shown in Figure 2, where the white areas are not labelled.

### 2.2. Problem Formulation

#### 2.2.1. Classification Problem

The main task in this paper for the aforementioned HSI is supervised classification. It can be formulated as follows. Let S={1,⋯,n} denote the set of HSI pixels, $x=\left(x_{1};\cdots ;x_{n}\right)\in R^{n\times d_{s}}$ is the corresponding pixel vector with *d* the number of bands. Let L={1,⋯,k} be a set of class labels and $C=(c_{1},\cdots ,c_{n})$ be the classification map corresponding to the label. Given a set of labelled data $T=\{\left(x_{1},c_{1}\right),\cdots ,\left(x_{\tau },c_{\tau }\right)\}$ with τ the total number of training samples, the objective of classification is to derive a classification mapping *C*, which assigns a label $c_{i}\in L$ to each pixel i∈S. In this paper, the following three widely used metrics [25] are adopted to evaluate classification performance:

(1) Overall Accuracy (OA): the percentage of correctly classified pixels;
(2) Average Accuracy (AA): the mean of the percentages of correctly classified pixels for each class;
(3) Kappa coefficient: the percentage of correctly classified pixels corrected by the number of agreements that would be expected purely by chance.

#### 2.2.2. SVM with Kernel

Due to its promising performance and effectiveness in coping with a small size of dataset, SVM is chosen as the baseline algorithm. SVM is a supervised non-parametric statistical learning technique, and consequently no particular assumption is made on the underlying data distribution [26]. Given a training set $T=\{\left(x_{i},c_{i}\right)|1\leq i\leq \tau \}$, they can be projected into a Hilbert space H (higher than the original feature space) using a proper mapping Φ(.) resulting in $T=\{\left({\left(\Phi \left(x\right)\right)}_{i},c_{i}\right)|1\leq i\leq \tau \}$. The SVM separates the data by using an Optimal Hyperplane $H_{p}$, which is determined by jointly maximizing the margin 2/||w|| and minimizing the sum of classification errors $\sum_{i=1}^{\tau }\xi_{i}$ under the constraint $c_{i}\left(x_{i}w+b\right)-1\geq 0,1\leq i\leq \tau$, as follows:

(1) $$\Psi \left(w,\xi \right)=\frac{1}{2}{\left||w|\right|}^{2}+K\sum_{i=1}^{\tau }\xi_{i},$$

where ξ’s are the so-called slack variables to account for data non-separability, and constant *K* is regularization parameter controlling the penalty assigned to errors and consequently can effectively control the shape of decision boundary. The optimization problem can be solved by considering the dual optimization through the use of Lagrange multipliers $\alpha_{i}$:

(2) $$\left\{\begin{array}{l} \max_{\alpha }:\sum_{i=1}^{\tau }\alpha_{i}-\frac{1}{2}\sum_{i,j=1}^{n}\alpha_{i}\alpha_{j}c_{i}c_{j}{\langle \Phi {\left(x\right)}_{i},\Phi {\left(x\right)}_{j}\rangle }_{H}\\ \mathrm{subject}\;\mathrm{to}\;\sum_{i=1}^{\tau }\alpha_{i}c_{i}=0,0⩽\alpha_{i}⩽K,\forall i\in \left[1,\tau \right]. \end{array}\right.$$

To avoid computing the inner products in the transformed space ${\langle \Phi {\left(x\right)}_{i},\Phi {\left(x\right)}_{j}\rangle }_{H}$, kernel function K is introduced in [27] so that ${\langle \Phi {\left(x\right)}_{i},\Phi {\left(x\right)}_{j}\rangle }_{H}=K\left(x_{i},x_{j}\right)$. The decision rule is finally given by

(3) $$f\left(x\right)=sign(\sum_{i=1}^{N_{s}}c_{i}\alpha_{i}K\left(s_{i},x\right)+b),$$

where $s_{i},1\leq i\leq N_{s}$ denote the support vectors. Different kernels leads to different SVMs, where the commonly used are polynomial kernel of order *p*, $K_{poly}\left(x,z\right)={\left(\langle x,z\rangle +1\right)}^{p}$ and Gaussian kernel $K_{gauss}\left(x,z\right)={exp(-\gamma ||x-z||}^{2})$ with γ being a parameter inversely proportional to the width of the Gaussian kernel [28]. Matlab function “fitcecoc” with “templateSVM” for algorithm setting is available to implement the SVM algorithm. Different mechanisms are available for multiclass classification, in this paper, one-vs-one (or all-vs-all) is adopted due to its simplicity and effectiveness [29].

### 2.3. Motivations Via Band Analysis

In this part, data analysis is performed on HSI data to shed light on its internal data properties, including correlation analysis between any two bands and sample bands along with box plot against different classes. On this basis, the research motivations of this paper are highlighted.

#### 2.3.1. Correlations between Bands

**Figure 3:**
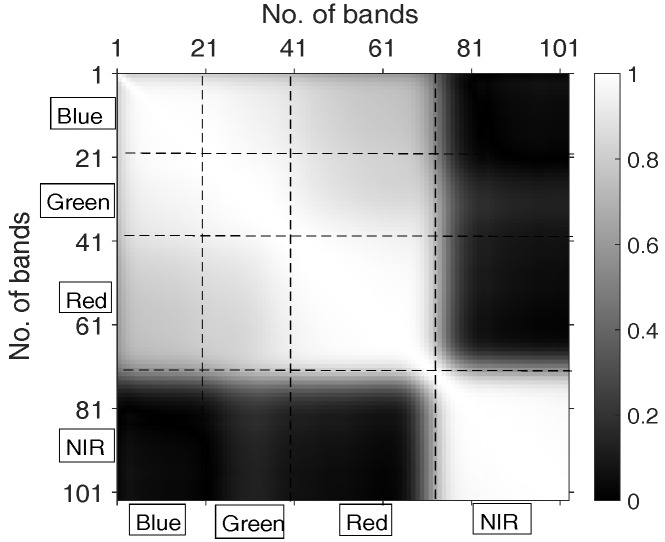
Absolute value of correlation matrix: lines are to separate different channels.

Pearson Correlation Coefficient (PCC) is a measure of linear correlation between two random variables [14]. For a pair of random variables (X,Y), it is defined by the formula

$$\begin{array}{c} r=\frac{\sum_{i}\left(x_{i}-\bar{x}\right)\left(y_{i}-\bar{y}\right)}{\sqrt{\sum_{i}\left(x_{i}-\bar{x}\right)}\sqrt{\sum_{i}\left(y_{i}-\bar{y}\right)}}, \end{array}$$

where $\bar{x},\bar{y}$ denote the mean of X,Y. Its value indicates the strength of the association, where 0 means no linear correlation, 1 (or –1) means totally linear positive (or negative) correlation. For a matrix $X_{n\times d}$, where *n* denotes the number of samples and *d* denotes the dimension of HSI bands, one can use Matlab command “corr” to calculate PCC between each pair of columns. The absolute value of the correlation matrix (element-wise) is plotted in Figure 3, where a brighter color indicates a higher value. It can be qualitatively seen from Figure 3 that

(1) Bands that are spectrally “near” to each other (i.e., close to matrix diagonal) tend to be highly correlated (brighter);
(2) There is an obvious dark rectangle (x-coordinate: bands No. 70–No. 103, y-coordinate: bands No. 1–No. 70), this is because bands No. 1 to No. 70 correspond to wavelength interval 430 nm to 700 nm (i.e., RGB visible light region) while No. 70 to No. 103 are wavelength interval 700 nm to 860 nm (i.e., NIR region), and the correlations between visible light and NIR are quite low;
(3) There are some grey areas showing the correlations for Red/Green, Red/Blue, Green/Blue, where the correlations of Red/Green and Red/Blue are slightly lower than Green/Blue. There are also four “bright” squares showing the correlations of bands within different channels.

To be more accurate, based on a total of $C_{103}^{2}=5253$ PCCs between different bands, we also present the histogram and empirical Cumulative Distribution Function of their absolute values in Figure 1.

It can be seen from Figure 1 that a large number of PCCs have absolute values larger than 0.7, taking up about 56%. That means many bands in HSI data are highly correlated, which may degrade classification performance if all bands are adopted in classification.

#### 2.3.2. Sample Bands with Box Plot

**Figure 4:**
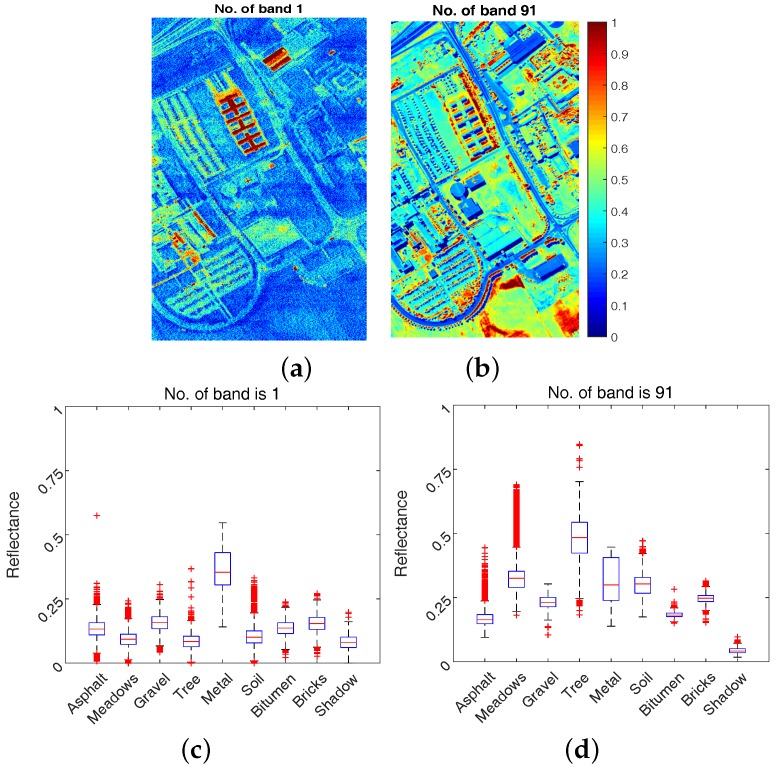
Band No. 1 (**a**) and No. 91 (**b**) with corresponding box plot analysis (**c**,**d**).

Some sample bands (No. 1 and No. 91) of HSI along with box plot (Matlab command “boxplot” is adopted) against different classes are displayed in Figure 4. In the box plot, five numbers are given including minimum, first quartile, median, third quartile, and maximum from bottom to top respectively, where the red dots are deemed to be outliers. First, it can be visually seen from the upper plots of Figure 4 that band No. 1 is more dominated by sensor noises (i.e., salt-and-pepper noise) in comparison to band No. 91. Second, it can be qualitatively seen from the lower plots of Figure 4 that the discriminating ability of band No. 1 and band No. 91 is different where band No. 91 is better, for example, it is easier to discriminate class shadow from others (e.g., Bricks, Metal, Soil) via band No. 91. More information on discriminating ability of different bands will be given in Section 4.1.1.

#### 2.3.3. Motivations

Based on the aforementioned analysis along with some practical considerations, the characteristics of HSI classification are summarized:

1. In HSI data, there are a large number of bands (e.g., 100 bands or more), where different bands have various differentiating ability in classification (see, Figure 4);
2. In these large number of bands, there exist high band redundancies (see, Figure 1), particularly in adjacent bands as shown in Figure 3;
3. When the dimension of features is high, generally large volume of training data are required, which may not be realistic in some applications such as crop health monitoring, since in such fields, it is hard, expensive and time-consuming for experts to collect comparatively sufficient labelled data, particularly for early disease detection;
4. With an increased number of features, the model parameter estimation of classification algorithms becomes very difficult as discussed in Section 1.
5. Training and classification time for algorithms with a large number of features is generally high.

Based on these observations, this paper investigates HSI classification with a limited training dataset for remote sensing applications. We approach this challenges by preparing an appropriate feature vector. Particularly, feature selection and feature extraction techniques in machine learning are adopted, which are introduced in Section 3.

## 3. Feature Selection and Extraction

Considering the fact that engineering a good feature space is of paramount importance to achieve high performance in many machine learning tasks. This section elaborates some typical feature selection and extraction approaches related to the HSI classification tasks in this paper.

### 3.1. Feature Selections

Feature selection is to identify a subset of original features so that good classification results can be achieved. On the one hand, reducing the number of features can reduce overfitting and improve model generalization. On the other hand, a better understanding of the features and their relationship with response variables can be gained. Suppose the complete feature set is *F*, where |F|=d is the number of features in *F*, feature selection is to optimally choose a subset S⊆F with |S|=m according to certain criterion. It should be noted that there are possible $2^{\left|F\right|}-1$ subsets, making exhaustive search of the best subset almost impossible due to the NP hardness.

All practical feature selection algorithms apply certain heuristics to guide the search process. The existing approaches can be loosely classified into three categories including filters, wrappers and embedded methods [30]. In filter approaches (e.g., MI [31]), the relevance between features and class label is chosen as the evaluating criterion, consequently independent of the classier used. While in wrapper approaches (e.g., Sequential Backward Elimination (SBE) [32]) , the overall classification performance is chosen as the criterion to include/eliminate a feature. It can be seen that wrapper approaches rely on training and testing a large number of classifiers, and filter approaches rely on the general characteristics of training data with independence of any classifiers [33]. Therefore, wrapper approaches are not considered in this paper due to their very high computation load. Besides, the so-called embedded methods (e.g., SVM-Recursive Feature Elimination [21]) are not included either, since they are constrained to certain classifier. For ease of understanding of filter based feature selection process, a diagram is drawn as follows.

**Figure 5:**
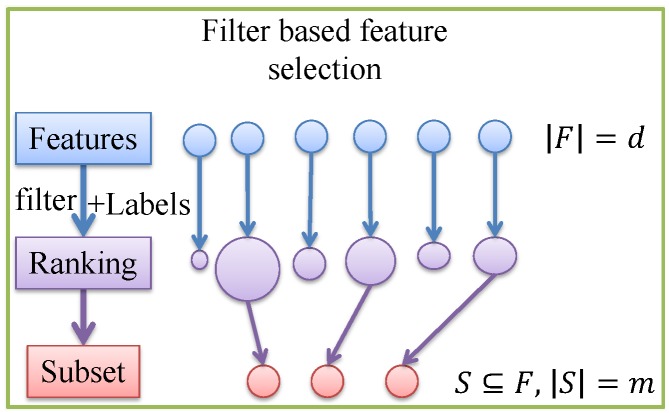
Diagram of filter based feature selection algorithm.

As depicted in Figure 5, the relevance between individual feature and class label is evaluated using different filter algorithms generating a feature ranking list. Features with high scores are then chosen as feature subset. In this paper, two different filter based feature selection approaches are considered including MI and Minimal-Redundancy-Maximal-Relevance (MRMR), which are detailed as follows.

#### 3.1.1. MI Approach

Feature scoring algorithms generate a score value for each feature to reflect its usefulness in classification. Several feature scoring algorithms are available according to different criteria [34] including MI [31], Fisher score [34], ReliefF and their variants. In this paper, MI [35] is adopted due to its computational efficiency and simple interpretation, which measures the statical dependence between two random variables or the amount of information that one variable contains about the other. In this approach, MI between individual feature (e.g., spectral band) and class label is examined, where a higher MI implies a higher relevance regardless of the classification algorithms. The MI between two discrete random variables *Y* and *Z* are defined by

(4) $$MI\left(Y,Z\right)=\sum_{y\in Y}\sum_{z\in Z}p\left(y,z\right)log\left(\frac{p(y,z)}{p(y)p(z)}\right)$$

where p(y,z) is the joint probability distribution function of *Y* and *Z*, and p(y) and p(z) are the marginal probability distribution functions of *Y* and *X* respectively. In MI approach, features are first ranked by their importance represented by MI scores, and the ones with high score are put together resulting in the feature subset of an appropriate dimension.

*Remark:* It should be noted that MI is generally calculated for integer variables. In HSI data, the class label is an integer, however, the bands are continuous value between 0 and 1. To make the algorithm applicable, feature quantization is usually adopted to discretize continuous bands into discrete bins. In this paper, the quantization approach in [36] is adopted, which can make sure that each bin has approximately equal number of samples in the training set.

#### 3.1.2. MRMR Approach

It should be noted that in aforementioned MI scoring approach, the dependence between selected features has not been considered, which may result in redundant features and degrade classification performance. To this end, MRMR approach developed in [37] is further considered. MRMR not only considers MI between features and class label to maximize feature relevance but also considers MI among the discretized features in the selected feature set so that feature redundancy can be minimized.

Similar to MI approach, the continuous-valued features need to be first quantized into different levels by placing quantization boundaries. In this paper, the default quantization approach in original MRMR is adopted, which places quantization boundaries at μ±σ where μ and σ denote the feature’s estimated mean and standard deviation respectively. After the discretized features y(f),f∈F are resulted, one can select the features based on the following role:

(5) $$S_{d}=S_{d-1}\cup \underset{f}{\mathrm{argmax}}\;\left[MI\left(y\left(f\right),z\right)-\frac{1}{d-1}\sum_{g\in S_{d-1}}MI\left(y\left(f\right),y\left(g\right)\right)\right]$$

where MI is defined in Equation (4), and *z* is a categorical variable representing class label. The remaining procedures are the same as that of MI approach.

### 3.2. Feature Extractions

#### 3.2.1. Principal Components Analysis

Principal Components Analysis (PCA) [19] is a powerful technique for dimensionality reduction and feature extraction. In standard PCA, a set of possibly correlated features are projected into new space represented by the unit orthogonal eigenvector basis, where coordinates in the new basis are called principal components (PCs). The transformation matrix is chosen so that the first PC has the largest possible variance, and each succeeding component in turn has the highest variance possible.

Mathematically speaking, suppose *x* is a vector of *d* random variables, $C_{x}$ is its covariance or sample covariance matrix. PCA constructs an orthogonal transformation y=Ax with $A=\;[u_{1}^{T};\cdots ;u_{d}^{T}]$, where $u_{k}^{\prime }s$ with k∈[1,d] are the unit orthogonal eigenvectors of matrix $C_{x}$ satisfying

$$\begin{array}{c} C_{x}u_{k}=\lambda_{k}u_{k}, \end{array}$$

where $\lambda_{k}^{\prime }s$ are the eigenvalues of matrix $C_{x}$, satisfying $\lambda_{1}\geq ,\cdots ,\geq \lambda_{d}.$ It can be shown that $u_{k}^{\prime }s$ can maximally explain the variance $Var\left(u_{k}^{T}x\right)=u_{k}^{T}C_{x}u_{k}$ with maxima $\lambda_{k}$.

#### 3.2.2. Kernel PCA

**Figure 6:**
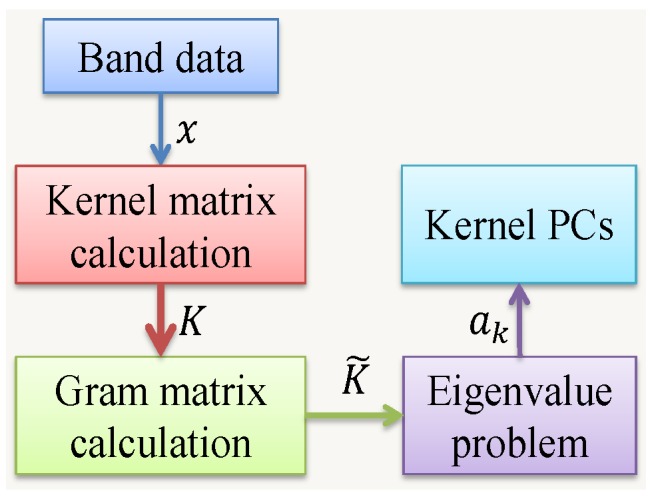
Flowchart for Kernel PCA.

Standard PCA only allows linear dimension reduction by performing a linear transformation. For data with more complicated structures, which may not be well represented in linear subspaces, kernel PCA in [38] may work more effectively using a nonlinear transformation with kernel methods for the sake of computation simplicity. The flowchart for kernel PCA and detailed steps are given in Figure 6 and Algorithm 1, respectively.

**Algorithm 1** Steps for Kernel PCA
(i)Construct the kernel matrix *K* from training dataset using $K_{i,j}=K\left(x_{i},x_{j}\right)$;(ii)Compute Gram matrix $\tilde{K}=K-1_{n}K-K1_{n}+1_{n}K1_{n}$, where $1_{n}$ is the n×n matrix with all elements equal to 1/n [39];(iii)Solve the eigenvalue problem $\tilde{K}a_{k}=\lambda_{k}na_{k}$, where $a_{k}={\left[a_{k1},\cdots ,a_{kn}\right]}^{T}$;(iv)Compute the kernel PCs $y_{k}\left(x\right)=\sum_{i=1}^{n}a_{ki}K\left(x,x_{i}\right)$.


As discussed in Section 2.2.2, different kernels are available such as polynomial kernel and Gaussian kernel. In the paper, the existing Matlab code in [40] is adopted to perform kernel PCA dimension reduction with polynomial kernel.

## 4. Experimental Results and Discussions

**Figure 7:**
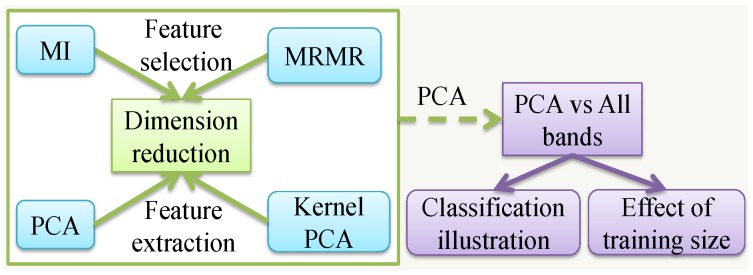
Flowchart of experimental verification.

In this section, different dimension reduction techniques are first implemented and their performance is compared. A further study is conducted to demonstrate the advantages of incorporating dimension reduction techniques in HSI classification. Particularly the effect of the size of training data is evaluated in detail. The flowchart of this section is given in Figure 7.

### 4.1. Feature Selection vs. Feature Extraction

#### 4.1.1. Feature Selection

**Figure 8:**
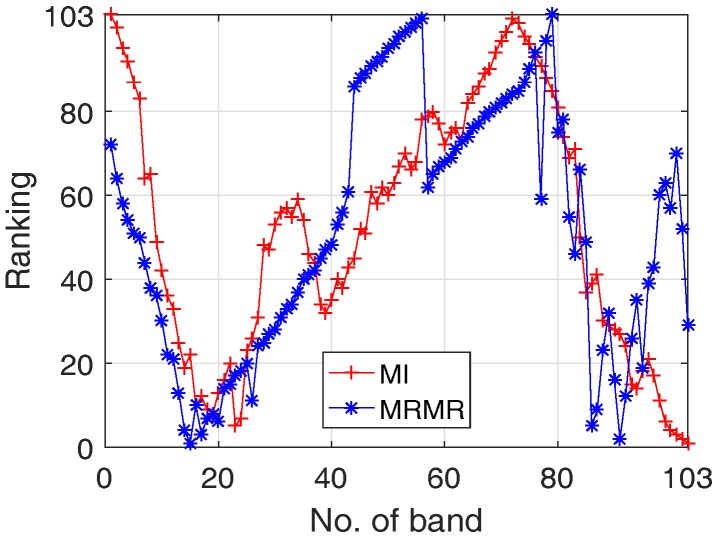
Ranking of each band using MI (red+ line) and MRMR (blue* line), where the x-axis is the band number and y-axis denotes the ranking where smaller value means higher ranking.

Comparative results between MI and MRMR based feature selection approaches are presented. It should be noted that in both approaches the discretized band using quantization and class label (integers 1–9 is used to represent different classes) are treated as discrete-time random variables. 2% of the labelled data are chosen as the training data. The rankings of each band using different approaches are depicted in Figure 8, where ranking with a small value implies a higher relevance.

It can be seen from Figure 8 that both approaches indicate that the most significant bands are in the areas around band No. 20 and No. 100, although for a particular band, its ranking is different due to different criteria adopted, where MI only considers the relevance between feature and class label, and MRMR considers feature/class relevance and feature redundancy simultaneously.

**Figure 9:**
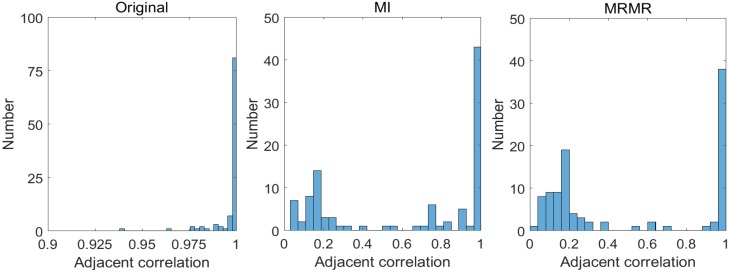
Correlation histogramsfor adjacent bands in original band order and rearranged band orders.

Correlation between adjacent bands in original band order and rearranged band order using MI and MRMR band ranking algorithms are shown in Figure 9. It can be seen that in original band order (i.e., left histogram), the correlation between adjacent bands is quite high (all above 0.925). Both feature ranking approaches (i.e., middle and right) can mitigate this phenomenon. However, MRMR approach directly considers feature redundancy in feature ranking and so outperforms MI approach, where 57 out of 102 correlation values are less than 0.45 in MRMR, and the value for MI is 39.

**Figure 10:**
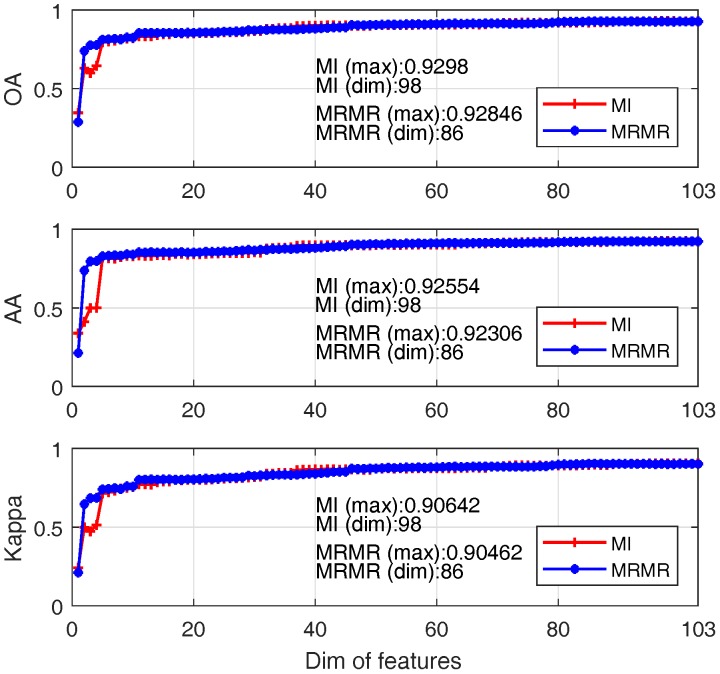
Comparative results betweenMI and MRMR: MI (red+ line); MRMR (blue* line).

Classification performance between two feature selection aided SVM are compared in terms of OA, AA and Kappa coefficient. The results are shown in Figure 10, where x-axis denotes the first *i* bands used and y-axis denotes the performance values of different metrics.

One can see that:

(1) For both approaches, the classification accuracy reaches 80% when about first 10 significant bands are adopted;
(2) When band number is less than 10; MRMR outperforms MI for most cases; this is mainly due to the better features produced by MRMR by considering both feature relevance and redundancy;
(3) The best OA of MI and MRMR based SVM are 93% and 92.8% with band numbers 98 and 86.

#### 4.1.2. Feature Extraction

**Figure 11:**
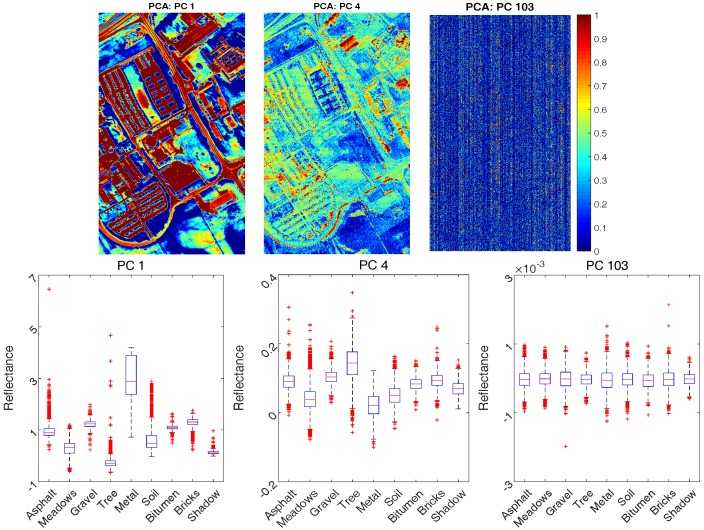
Sample PC bands (PC 1, PC 4 and PC 103) of linear PCA, and the corresponding box plot for the labelled data.

Performance of feature extraction aided classification algorithms including PCA based SVM and Kernel PCA based SVM is evaluated. Particularly, in Kernel PCA, polynomial kernel with order two is chosen after trial and error testing. PC bands with smaller eigenvalues contain less information about data variance, and most of the information is contained in the first few [41]. Sample PCs of linear PCA along with box plot against different classes are plotted in Figure 11 as an illustrating example. It can be visually seen from the upper plots of Figure 11 that PC 1 contains more information than PC 4 and PC 103, and PC 103 is mainly dominated by noises compared with others. As a result, their discriminating ability is very different as shown by the lower plots of Figure 11. It is easier to differentiate different classes by using PC 1, and there is almost no discriminating ability in PC 103 since there is little difference in the distributions of different classes by using PC 103.

**Figure 12:**
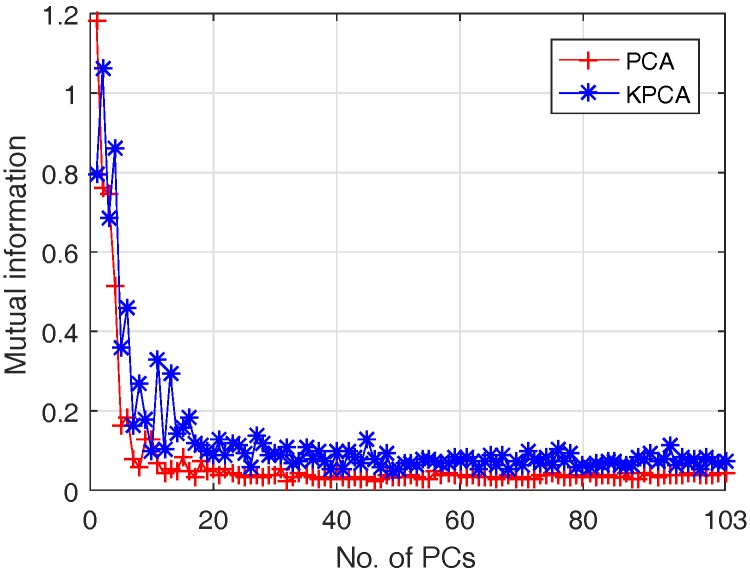
MI between each PC and class label for PCA and KPCA: PCA (red+ line); KPCA (blue* line).

To quantitatively evaluate the usefulness of different PCs in classification, MI between each PC and class label is calculated for both approaches, which is plotted in Figure 12.

**Figure 13:**
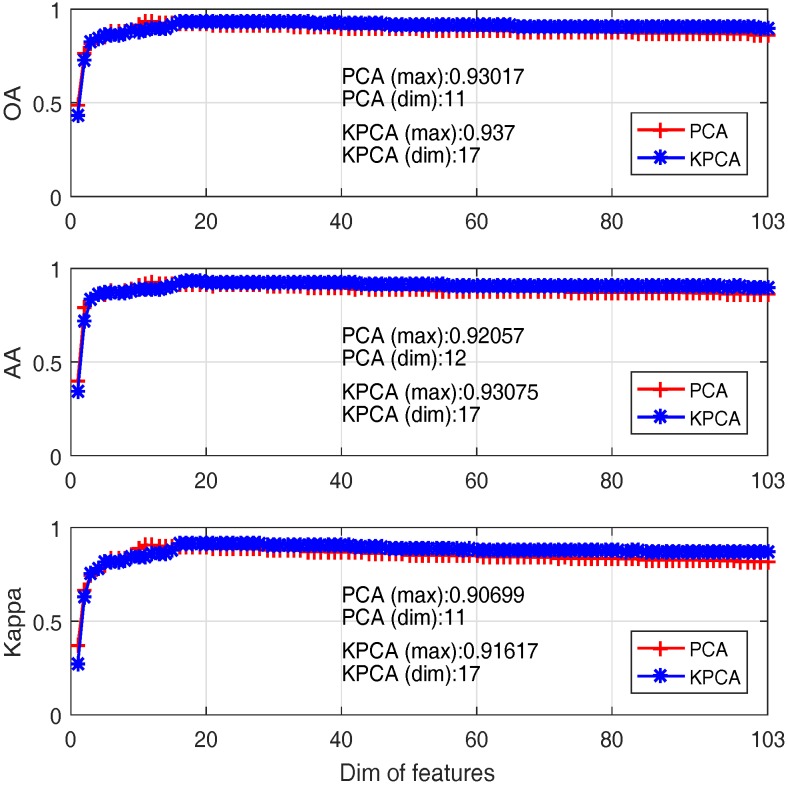
Comparative results between PCA (red+ line) and Kernel PCA (blue* line) based approaches.

It can be seen from Figure 12 that only the first several PCs have a high MI value.That means only the PCs with high MI value are required for classification rather than using all the PCs. Classification performance of PCA and Kernel PCA based SVM under different numbers of first PCs as feature vector are compared and presented in Figure 13, where x-axis denotes the first *i* PCs adopted and y-axis denotes the performance values of different metrics.

One can see that:

(i) Classification accuracy reaches 80% when only the first 3 PCs are adopted, although the first 3 PCs account for 99% of the cumulative variance; this is because data variance is not exactly a good reflection of the data structure when it comes to classification;
(ii) The best OA of PCA and Kernel PCA are 93% and 93.7%, when the number of bands are 11 and 17 respectively. That means Kernel PCA obtains similar (or slightly better) performance than PCA, but require a larger number of PCs.

#### 4.1.3. Feature Selection vs. Feature Extraction

Comparisons between feature selection and feature extraction based dimension reduction techniques are further performed. The aspects for comparison comprise: (1) all bands information required or not; (2) their performance comparisons; (3) the number of features to reach best performance. The results are summarized in Table 1.

One can conclude that:

(1) Only selected bands are needed in MI and MRMR approaches, however, all original bands are needed in PCA and Kernel PCA so that new features can be extracted;
(2) The best performance of all four algorithms are quite close (no statistical difference);
(3) To achieve the best performance, generally a larger number of features are required in MI and MRMR based feature selection approaches in comparison with PCA and Kernel PCA based feature extraction approaches;
(4) Most importantly, linear PCA based approach is recommended since it is relatively simple, with low computational cost and a smaller number of features required to achieve its best performance.

### 4.2. PCA vs All Bands

#### 4.2.1. Classification Illustration

**Figure 14:**
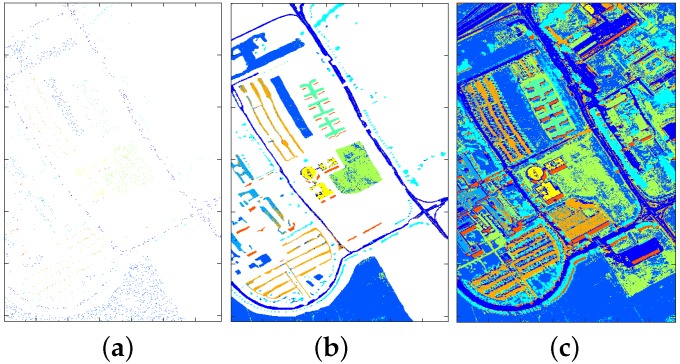
Classification results for SVM with all bands: training data (**a**); classification map for all labelled area (**b**); classification map for whole image (**c**).

**Figure 15:**
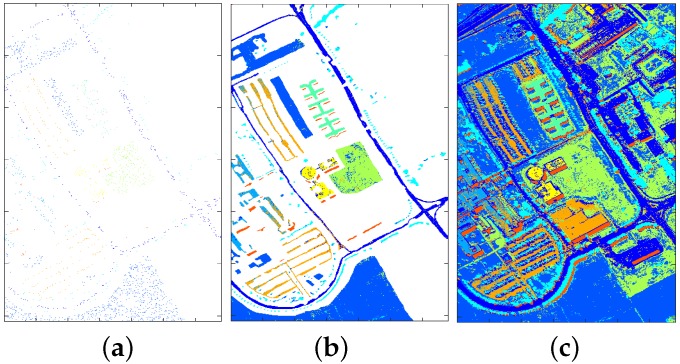
Classification results for SVM with PCA: training data (**a**); classification map for all labelled area (**b**); classification map for whole image (**c**).

In this section, the advantages of dimension reduction for classification are demonstrated where SVM is chosen as the baseline algorithm. Following the conclusions from Section 4.1.3, PCA with 11 features is chosen as dimension reduction technique. Specifically, the comparative results between SVM with PCA and SVM with all bands are presented, where the size of training samples is 2% of the labelled data and the remaining 98% is used for testing. For the purpose of illustration, the trained classifiers are applied to the labelled area and the whole HSI image respectively. The classification maps generated by SVM with all bands and SVM with PCA are shown in Figure 14 and Figure 15 respectively.

**Figure 16:**
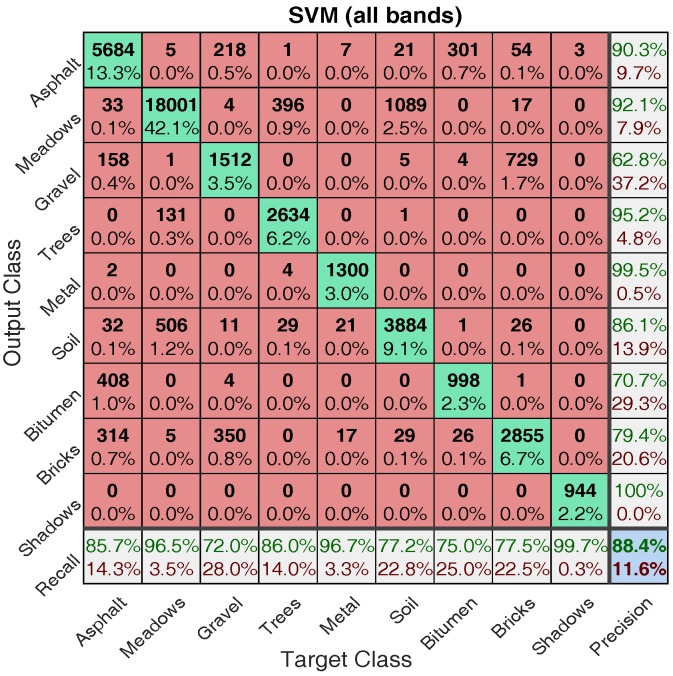
Confusion matrix for SVM with all bands.

**Figure 17:**
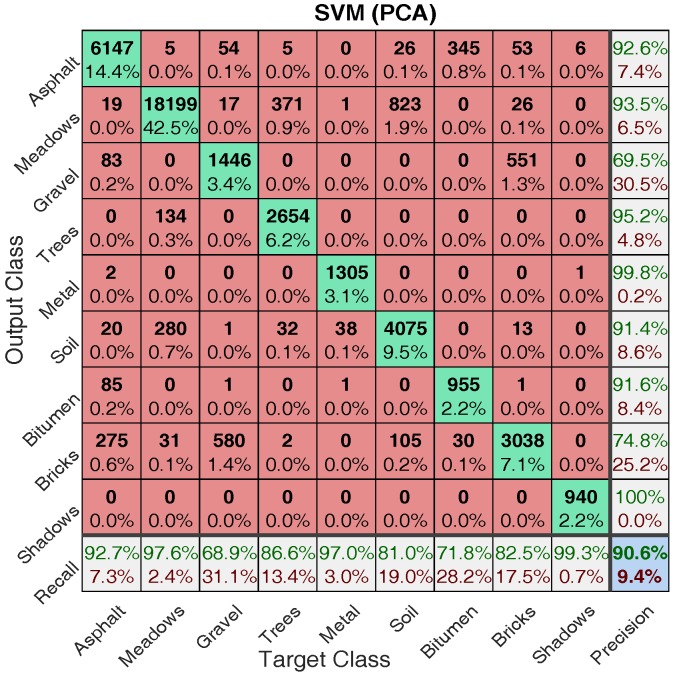
Confusion matrix for SVM with PCA.

Comparing the classification maps in Figure 14 and Figure 15 with ground truth map in Figure 2, one can see that reasonably accurate classification have been achieved by both approaches. To make a more detailed comparison, confusion matrices for both approaches are calculated and shown in Figure 16 and Figure 17, where target class denotes ground truth and output class denotes predicted class.

In this figure, the diagonal cells in green show the number and corresponding percentage of correct classification for different classes. Taking Figure 16 as an example, 5684 Asphalt samples are correctly classified as Asphalt corresponding to 13.3% of all 42,776 samples. The off-diagonal cells show where mistakes occur. For example, in the first row, 218 Gravel samples are incorrectly classified as Asphalt corresponding to 0.5% of all 42,776 samples. The rightmost column presents the accuracy for each predicted class (termed precision), while the bottom row shows the accuracy for each true class (termed recall). For example, out of 5684 + 5 + 218 + 1+ 7 + 21 + 301 + 54 + 3 = 6294. Asphalt prediction, 90.3% (5684) are correct prediction and 9.7% are wrong prediction; out of 5684 + 33 + 158 + 2 + 32 + 408 + 314 = 6631. Asphalt samples, 85.7% (5684) are correctly predicted as Asphalt and 14.3% are wrongly predicted as others. The cell at the right bottom shows the overall accuracy (the ratio between the summation of diagonal numbers and all samples), which for SVM with all bands is 88.4%.

One can see from Figure 16 and Figure 17 that for training data with 2% proportion of all labelled data, SVM with PCA obtains better performance in terms of overall accuracy (88.4% → 90.6%), precision (except Gravel, Bitumen, and Shadows) and recall (except Bricks). Beside, the top three misclassified classes for SVM with all bands are: Soil → Meadows (2.5%), Brick → Gravel (1.7%), Meadows → Soil (1.2%); while the top three misclassified classes for SVM with PCA are: Soil → Meadows (1.9%), Gravel → Bricks (1.4%), Bricks → Gravel (1.3%). It is no surprise that it is hard to classify Meadows and Soil, since as can be seen from Figure 2 (bottom area) that the grass in Meadows is at an early stage and there exist bare Soil in Meadows. Another point worth mentioning is that, the classification time of SVM with PCA (about 0.68 s) is substantially smaller than that of SVM with all bands (about 3 s) considering the larger feature dimension in SVM with all bands. More information regarding computation load is provided in Section 4.2.2.

#### 4.2.2. The Effect of Training Size

We further investigate the effect of different numbers of training samples on classification performance. In HSI classification the size of training samples is generally small due to the high cost (in terms of time, money and manpower) of acquiring large volume of groundtruth data. To fully assess their behaviours, the performance of these two SVM algorithms is evaluated under different size of training dataset by choosing the training dataset in the range 0.5% to 15% (the remaining labelled samples are testing datasets) of the whole labelled ROSIS. With trained SVM models, their classification performance and computation time are calculated by using all labelled ROSIS data. 200 simulations are run to account for the randomness, where the samples for training in each run are randomly selected.

**Figure 18:**
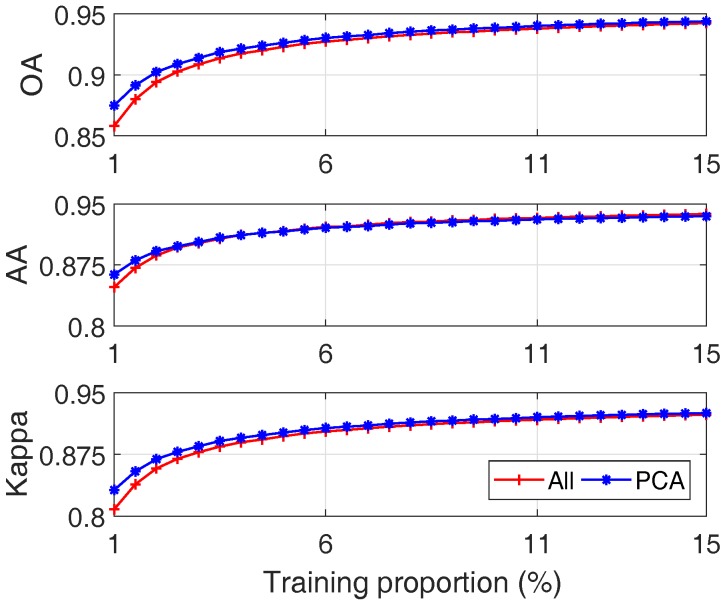
Comparative resultsbetween SVM with PCA (blue* line) and SVM with all bands (red+ line). x-axis denotes different proportions of training samples.

The comparative results in terms of OA, AA and Kappa coefficient (mean value) are shown in Figure 18, where their average values are plotted.

**Figure 19:**
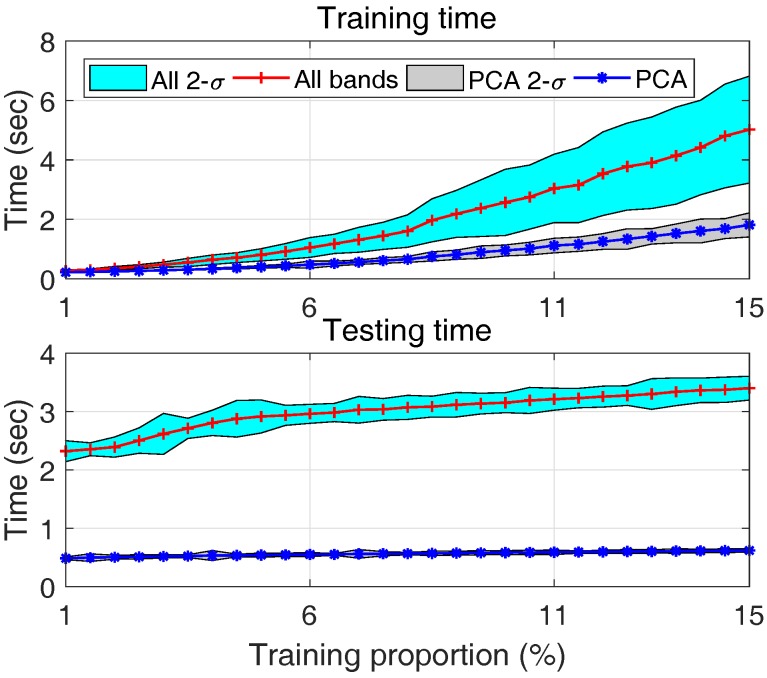
Comparative results of computation time under different sizes of training dataset including training time (upper plot) and testing time (lower plot): mean (red+ line) and 2-σ region (green area) of all bands, mean (blue* line) and 2-σ region (gray area) of PCA.

The corresponding computation time is shown in Figure 19, where both mean value and 2-σ regions are plotted.

The following conclusions can be drawn:

(1) With an increased number of training samples, both approaches can get better results, which is consistent with the common sense in machine learning;
(2) When the size of training data is small, both approaches degrade; this is due to the sparseness of training data. However, SVM with PCA outperforms SVM with all bands as shown by the left part of the plots of Figure 18. This is because by reducing the number of features using PCA, the effect of data sparseness can be attenuated to some extent.
(3) With an increased size of training dataset, the training time for both approaches increases as shown by the upper plot of Figure 19. However, PCA based approach always requires less time, about 1/3 of all bands based approach. This is mainly due to the substantially reduced number of features in PCA based approach.
(4) This is even more significant in real-time classification, it can be seen from the lower plot of Figure 19 that the computation time of all bands approach is about 6 times that of PCA approach, and this ratio keeps increasing with more training samples. This is because, on the one hand given a number of training data the SVM model using all bands is more complex due to the larger number of features; on the other hand, given a number of testing samples, the SVM model trained using a larger number of training samples is usually more complex due to an increased number of support vectors. The testing time of PCA based approach, due to the reduced number of features, is less sensitive to the size of training dataset.
(5) Considering the classification performance (similar or even better) and computation load for both training (1/3) and testing (1/6), one can conclude that compared with SVM by using all bands, SVM with selected features using PCA is more appropriate for real-time applications, particularly when the training data are very limited.

**Figure 20:**
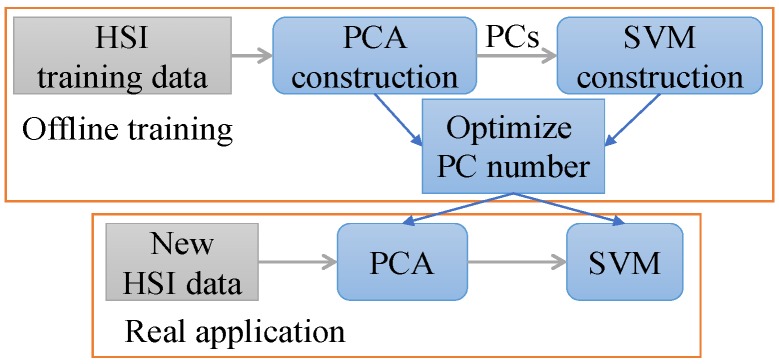
Flowchart of the PCA aided SVM for HSI classification.

The complete flow chart for the PCA aided SVM is displayed in Figure 20.

## 5. Conclusions and Future Work

In this paper, the problem of Hyperspectral image (HSI) classification with limited training data is investigated. To remove the irrelevant and redundant data, different approaches are elaborated and compared including feature selection (i.e., mutual information and minimal-redundancy-maximal-relevance) and feature extraction (i.e., Principal Component Analysis (PCA) and kernel PCA). Comparatively experimental results on real HSI dataset (i.e., ROSIS data) demonstrate that PCA is the most effective dimension reduction approach in term of classification performance, algorithm complexity and computation load. After identifying PCA as a promising feature extraction approach, we further compare SVM with PCA and SVM using all bands under various sizes of training data. Further experimental results demonstrate that by augmenting SVM with PCA, the obtained predictors provide better prediction performance in terms of overall accuracy, average accuracy, kappa coefficient when the dataset is of a small size and comparable performance when there are sufficient training data in comparison with classic SVM algorithms. In all the cases, significant less computational time (particularly in testing) is achieved which is important in facilitating real-time classification using HSI, particularly for platforms with limited computational resources such as unmanned aerial vehicles. Considering SVM with PCA requires few feature dimension while guaranteeing good performance with a small size of training data, it is quite appropriate for real remote sensing applications.

This paper mainly focused on maximally exploiting the potential of pixel-wise (or spectral) HSI classification with limited training samples using feature selection and extraction techniques. With the advent of HSI with higher spatial resolution by using Unmanned Aerial Vehicles (UAVs) as the camera platform, more information will be explored in the future such as spatial or even contextual information. It should also be highlighted that the feature selection and extraction techniques discussed in this paper can be parallelly applied to efficiently capture the spatial or contextual features. Besides, hybrid approaches by simultaneously exploiting feature selection and feature extraction techniques can also be considered to further improve the performance.

## Figures and Tables

**Table 1:** Comparisons between feature selection (FS) and feature extraction (FE).

#	MI	MRMR	PCA	KPCA
Type	FS	FS	FE	FE
All bands	No	No	Yes	Yes
Best OA	92.98%	92.85%	93.02%	93.70%
Best AA	92.55%	92.31%	92.06%	93.08%
Best Kappa	0.906	0.905	0.907	0.910
No. features	98	86	11	17
